# Experimental and Statistical Analysis of the Effect of Heat Treatment on Surface Roughness and Mechanical Properties of Thin-Walled Samples Obtained by Selective Laser Melting from the Material AlSi10Mg

**DOI:** 10.3390/ma16237326

**Published:** 2023-11-24

**Authors:** Sergey N. Grigoriev, Nikita Nikitin, Oleg Yanushevich, Natella Kriheli, Olga Kramar, Roman Khmyrov, Idarmach Idarmachev, Pavel Peretyagin

**Affiliations:** 1 Spark Plasma Sintering Research Laboratory, Moscow State University of Technology “STANKIN”, Vadkovsky per. 1, Moscow 127055, Russia; s.grigoriev@stankin.ru (S.N.G.); p.peretyagin@stankin.ru (P.P.); 2 Federal State Budgetary Educational Institution of the Higher Education, A.I. Yevdokimov Moscow State University of Medicine and Dentistry, Ministry of Healthcare of the Russian Federation, Moscow 127006, Russia; olegyanushevich@mail.ru (O.Y.); nataly0088@mail.ru (N.K.); dr.ovkramar@gmail.com (O.K.); 3 Laboratory of Innovative Additive Technologies, Moscow State University of Technology “STANKIN”, Vadkovsky per. 1, Moscow 127055, Russia; r.khmyrov@stankin.ru (R.K.); _idarmachev_@mail.ru (I.I.)

**Keywords:** selective laser melting, aluminum alloys, surface roughness, heat treatment, mechanical properties

## Abstract

Statistical analysis of mechanical properties of thin-walled samples (~500 microns) obtained by selective laser melting from AlSi10Mg material and subjected to heat treatment for 1 h at temperatures from 260 °C to 440 °C (step of aging temperature change 30 °C) has shown that the maximum strain hardening in the stretching diagram section from yield strength to tensile strength is achieved at the heat treatment temperature equal to 290 °C. At carrying out of correlation analysis, a statistically significant positive correlation between deformation corresponding to yield strength and the sum of heights of the largest protrusions and depths of the largest depressions of the surface roughness profile within the basic length of the sample (Rz) and the full height of the surface roughness profile (Rmax) was established. It was found that the reason for the correlation is the presence of cohesive states between the extreme values of the surface roughness profile that persist along the entire length of the specimen.

## 1. Introduction

One of the main directions of the development of modern industrial technologies is the creation of high-quality products with low production costs. Reduction of production costs can be achieved by reducing to the minimum possible time of creation of the final product-“from idea to finished product” with simultaneous preservation of high-quality manufacturing. Among the technologies actively introduced in the production process, additive manufacturing technologies fall under these requirements.

ISO/ASTM 52900:2015 classifies the technologies used in additive manufacturing and considers the type of raw materials, deposition techniques, and methods of melting or curing the material [1]. The most common technologies of additive manufacturing are SLA and FDM printing [2,3,4,5,6]; these technologies use thermoplastics and polymer resins as the main materials, which limits the scope of application of products made by these technologies. Technologies that allow manufacturing products from metal, such as selective laser melting (SLM) technology [7,8,9,10,11,12,13,14,15], have a wider industrial potential. Powders of metals and alloys of various compositions are used as a starting material to produce final products using selective laser melting technology. 

One of the most promising, from the industrial and environmental [16] point of view, materials for use in additive manufacturing are aluminum and aluminum-based alloys. The combination of low cost of aluminum and aluminum-based alloys, good mechanical properties, and additive technologies allows for a shortened production cycle of the final product and reduces the cost of the final product. 

Currently, the list of aluminum-based alloys used in the manufacture of products using additive technologies, including methods of selective laser melting, is quite extensive [17,18]. However, despite a large amount of research, there are still several problems that require consideration. Optimization of additive manufacturing parameters to obtain a product with specified structural properties, namely mechanical strength and surface roughness [17].

In the presented work, samples made by SLM technology from light alloy AlSi10Mg were studied.

AlSi10Mg has good mechanical strength and corrosion resistance [19,20,21,22] and allows the manufacture of products using SLM technology of complex geometric shapes [23]. Kamarudin et al. [23] note that during the manufacture of complex-shaped products (molds), inhomogeneity of surface roughness and deviation of actual dimensions from the design dimensions are observed, which is attributed to the influence of local heat transfer. Studies [24,25] show that the effect of local heat transfer affects the microstructure of the bulk product and, consequently, the mechanical properties. In addition, the change in mechanical properties of the final product depends on the tilt angle of the product during printing. Changing the tilt angle from 35.5° to 90° leads to an increase in mechanical properties by 12% (as the angle increases) while the surface roughness decreases [24].

An increase of mechanical properties at the manufacturing of specimens by SLM technology from AlSi10Mg material is achieved due to hardening. The main mechanism of hardening is precipitate hardening, which contributes more than the hardening of Si solid solution in the α-Al matrix [25]. Clarification of the mechanisms of mechanical properties enhancement of AlSi10Mg samples obtained by selective laser melting shows that precipitate strengthening is achieved due to a very thin Al-Si eutectic structure between α-Al dendrites and the formation of a microstructure oriented transversely to the direction of load application, and the anisotropy of properties becomes minimal when the scanning speed is optimized [26,27,28,29]. Additional sources of improvement of mechanical properties of the samples are changes in the gas atmosphere in which selective laser melting is performed, changes in surface roughness and porosity, and thermal post-treatment of parts manufactured by SLM printing technology from AlSi10Mg. When argon was replaced by nitrogen in the working chamber of the SLM 3D printer, the achievement of the strength limit of ~350 MPa was recorded [29].

The influence of porosity and surface roughness of the samples obtained by SLM printing technology from AlSi10Mg has received a great deal of attention [30,31,32,33,34,35,36,37,38,39,40,41,42,43,44,45,46,47]. The focus of the works is related to the optimization of technological parameters to reduce surface roughness and porosity and, consequently, to increase hardness, impact toughness, and fatigue strength by reducing the surface roughness of samples obtained by SLM printing technology from AlSi10Mg. In particular, the critical point of energy density, which gives the minimum pore fraction for AlSi10Mg and is about 60 J/m^3^ [41] and exposure time of 140–160 μs [42], was shown to exist. In addition to the optimization of technological parameters, the influence of different surface post-treatment methods on the mechanical properties of samples has been investigated [33,46]. It is noted in [33] that strong vibration hardening had the greatest effect on the improvement of fatigue life, followed by laser hardening and shot peening.

However, the works do not analyze the changes in tensile mechanical properties as a function of surface roughness on thin-walled samples, where the contribution of the surface to the tensile strength may be significant.

The influence of thermal post-treatment on the mechanical properties of samples produced by SLM technology is under active study [48] and requires detailed elaboration. In the works [31,46], the application of standard heat treatment T6 is considered, and it is shown that the average surface roughness of samples obtained by SLM technology from AlSi10Mg material decreased after heat treatment at 540 °C for 2 h. However, after artificial aging at 155 °C for 12 h and initially at 530 °C for 2 h, the surface roughness increased [34]. The lack of significant hardening of the material under the standard T6 heat treatment regime is also confirmed [49]. In [50], the occurrence of anisotropy of mechanical properties arising in horizontally annealed samples during heat treatment carried out at 270 °C for 1.5 h was demonstrated, and a decrease in properties compared to non-annealed samples was observed, indicating the need for further search for an optimal heat treatment regime.

The analysis shows that earlier, the connection between mechanical properties in the tension of thin-walled samples made by technology of selective laser melting of AlSi10Mg material (changes in the height of the surface profile is up to 40% of the thickness of the sample) and surface roughness has not been studied, as well as the question of choosing the optimum mode of heat treatment of thin-walled samples made by technology SLM of AlSi10Mg remains relevant.

Thus, the purpose of the presented work is to determine the effect of heat treatment at temperatures from 260 °C to 440 °C for 1 h on the tensile mechanical properties and surface roughness of thin-walled samples (~500 μm) manufactured by SLM technology from AlSi10Mg.

## 2. Materials and Methods

### 2.1. Mechanical Properties

The microstructure and chemical composition of the studied materials were analyzed using a Phenom ProX scanning electron microscope (Holland) equipped with an adapter for elemental analysis by energy dispersive spectroscopy. The surface roughness of the samples was measured using a HOMMEL-ETAMIC T8000 profilograph (JENOPTIK (Hommel-Etamic), Jena, Germany), mechanical tensile tests were performed on an INSTRON 5989 electromechanical testing machine (Instron, Norwood, MA, USA) at a speed of 2 mm/min and statistical analysis of the experimental results was performed using software (Rstudio 2023.06.1 Posit Software, PBC, GNU license) written in R language.

### 2.2. Production of Samples

**Figure 1:**
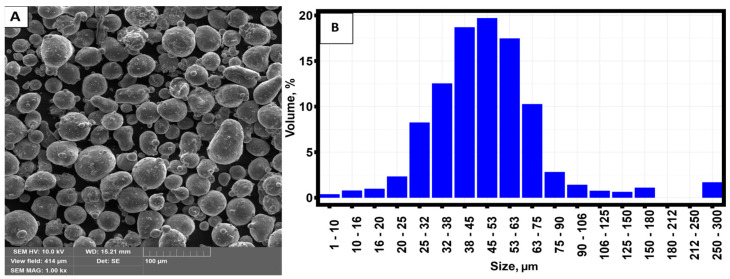
(**A**) SEM image of the initial AlSi10Mg powder. (**B**) Particle size distribution.

AlSi10Mg powder served as a starting material for the fabrication of samples by selective laser melting. The size of the powders ranged from 30 µm to 75 µm. Figure 1 shows a micrograph of the starting material and the size distribution of the powder particles.

The average chemical composition of the initial AlSi10Mg powder is presented in Table 1.

**Figure 2:**
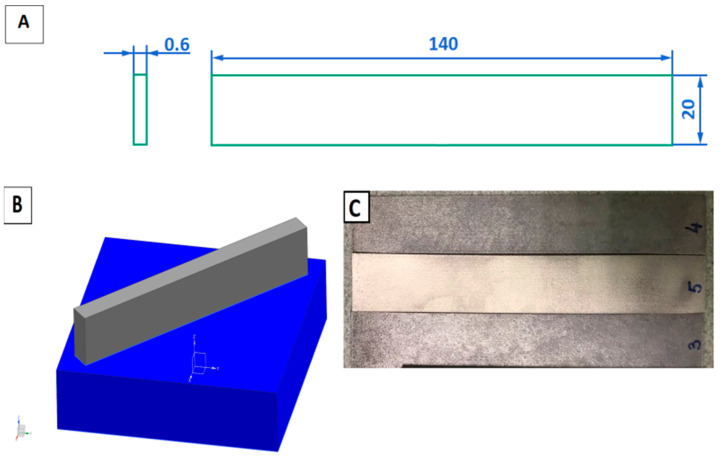
Schematic parameters of the sample made by selective laser melting method (**A**), its location on the table during printing (**B**), and samples made by selective laser melting method (**C**).

Printing was carried out on a Farsoon FS121M SLM selective laser melting machine (Farsoon Technologies, Changsha, China) with a pre-installed laser with a maximum power of 500 W. The main printing modes were layer thickness 30 µm, laser power P = 340 W, hatching distance 0.15 mm, and laser travel speed 1500 mm/s. Figure 2 shows a schematic drawing of the sample and its location on the table during fabrication by selective laser melting.

**Figure 3:**
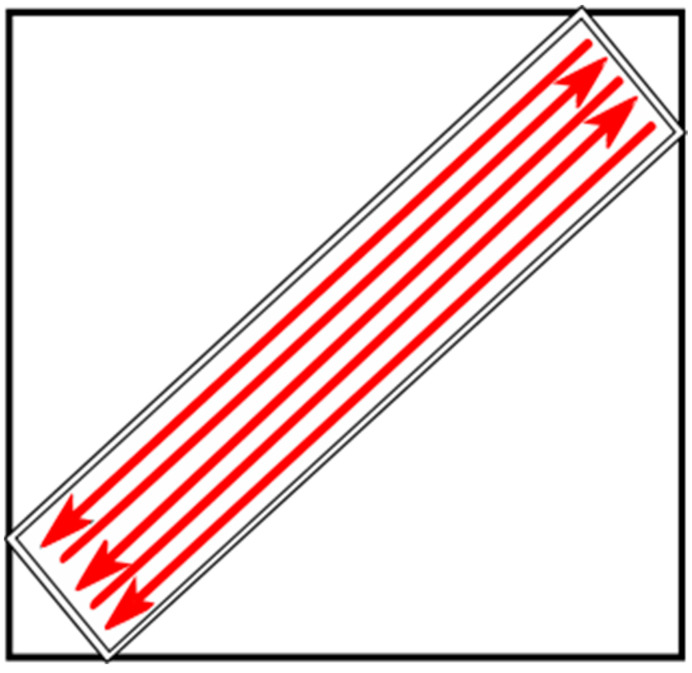
Schematic representation of the scanning strategy for fabrication of samples by SLM technology from AlSi10Mg.

AlSi10Mg samples made by selective laser melting technology were subjected to sandblast cleaning followed by heat treatment. The view of the samples after cleaning is shown in Figure 2C. Figure 3 shows the scanning strategy for the fabrication of samples using the SLM technique from AlSi10Mg.

Heat treatment of the samples was carried out in a muffle furnace in a natural atmosphere at temperatures from 260 °C to 440 °C with a step of 30 °C. The samples were heated up to the holding temperature with the natural heating rate of the furnace, held for 1 h at the set temperature, and cooled down with the natural cooling rate of the furnace. The heat-treated specimens were subjected to surface roughness analysis and mechanical testing. The geometry of the specimens subjected to tensile testing is shown in Figure 2A.

## 3. Results and Discussions

### 3.1. Surface Roughness of Samples Manufactured by Selective Laser Melting Technology from AlSi10Mg Material

A total of 48 samples made by selective laser melting technology from AlSi10Mg material were subjected to a surface roughness study. 

**Figure 4:**
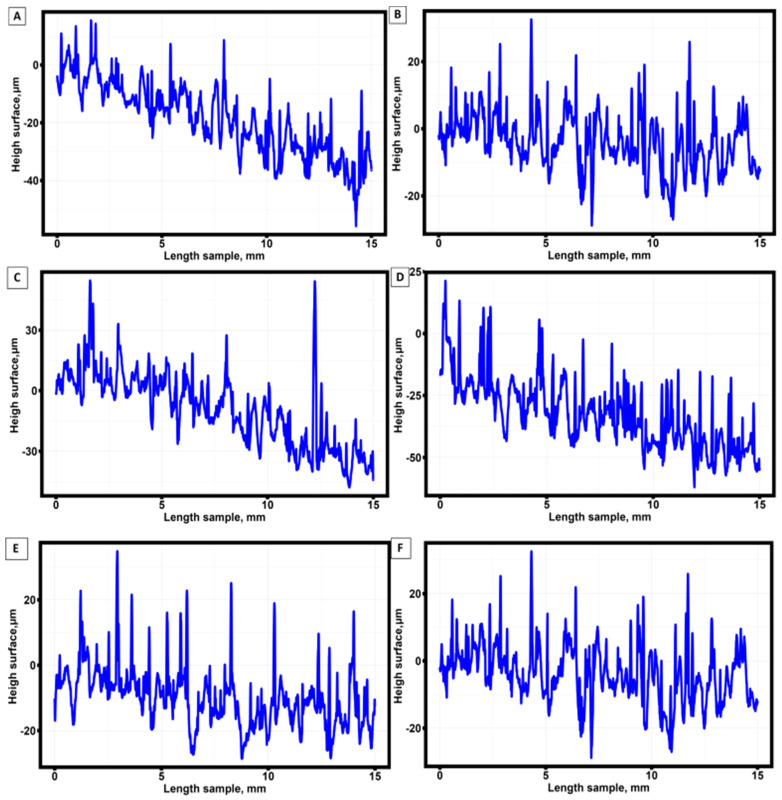
Surface profile for six unannealed samples. (**A**) sample No. 1; (**B**) sample No. 2; (**C**) sample No. 3; (**D**) sample No. 4; (**E**) sample No. 5; (**F**) sample No. 6.

Figure 4 shows the results of surface roughness profile measurements for the samples that were not annealed after fabrication by selective laser melting technology. Similar roughness diagrams were obtained for the other 42 samples.

The analysis of the autocorrelation functions of the surface profile shows that there is a regularity in the variation of the surface profile height depending on the sample length, which has the character of a stationary series [50].

Table 2 presents the arithmetic mean values of the absolute values of profile deviations within the base length (Ra), the sum of the height of the largest profile protrusion, and the depth of the largest profile depression within the base length of the sample (Rz) and the total profile height (Rmax) of all samples.

Table 3 presents the basic statistical analysis of the surface roughness parameters presented in Table 2.

The analysis of basic statistical characteristics shows that the arithmetic mean of absolute values of profile deviations within the basic length (Ra) does not have a wide scatter for different samples. At the same time, the greatest profile height, the sum of the height of the greatest profile protrusion, and the depth of the greatest profile depression within the basic length of the sample (Rz) have rather high fluctuations of values from sample to sample, the same behavior is observed for the total profile height (Rmax).

### 3.2. Mechanical Test Results for Groups of Specimens Manufactured by Selective Laser Melting Technology from AlSi10Mg Material, Pre-Treated at Different Temperatures

**Figure 5:**
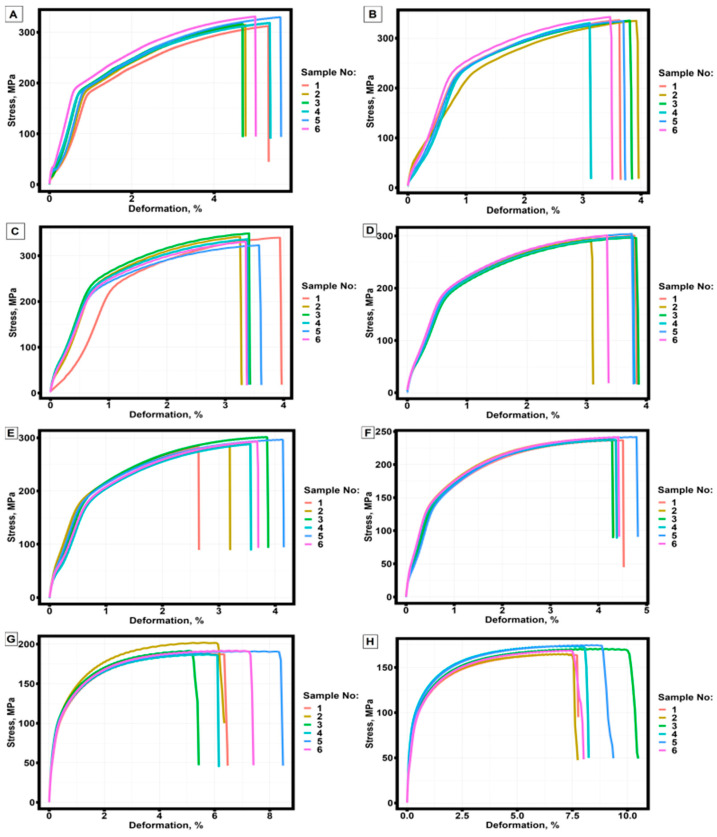
Engineering stress-strain curves of thin-walled samples obtained by selective laser melting technology from AlSi10Mg material with different annealing temperatures. (**A**) without annealing; (**B**) annealing at 260 °C; (**C**) annealing at 290 °C; (**D**) annealing at 320 °C; (**E**) annealing at 350 °C; (**F**) annealing at 380 °C; (**G**) annealing at 410 °C; (**H**) annealing at 440 °C.

A total of 48 specimens were subjected to tensile tests; 42 of them were pre-annealed at different temperatures. Figure 5 shows the engineering stress-strain curves of samples that did not undergo pre-annealing and samples that underwent pre-annealing at temperatures from 260 °C to 440 °C.

Table 4 presents the main mechanical properties of 48 tested samples.

Table 5 presents the basic statistical analysis of mechanical parameters presented in Table 4.

Preliminary analysis of the results of the basic statistical analysis shows that the maximum value of tensile strength and yield strength is achieved at annealing temperatures of 260 °C and 290 °C, while the maximum ductility is achieved at annealing temperature of 440 °C.

### 3.3. Statistical Analysis of the Results

The choice of the criterion for checking the experimental results belonging to the normal distribution is made based on calculations of the average statistical power depending on the number of tested samples. The average power of the criteria is calculated using the Monte Carlo method with the number of iterations equal to 100,000. During the calculations, a simple distribution was introduced into the criterion, the parameters of which were calculated by the maximum likelihood method. The Cauchy, exponential, Gumbel, log-normal, logistic, normal, and Weibull distributions were considered simple distributions. The distribution parameters were iteratively recalculated depending on the number of tested samples.

Four criteria were selected for the study:

- From parametric criteria:
  ○ Shapiro–Wilk criteria [51];
  ○ D’Agostino criteria [52];
- From non-parametric:
  ○ Kolmogorov–Smirnov criteria [53];
  ○ Anderson–Darling criteria [54];

Anderson–Darling criterion and D’Agostino criterion have limitations on the minimum number of studies; the number of studies must be greater than or equal to 7.

**Figure 6:**
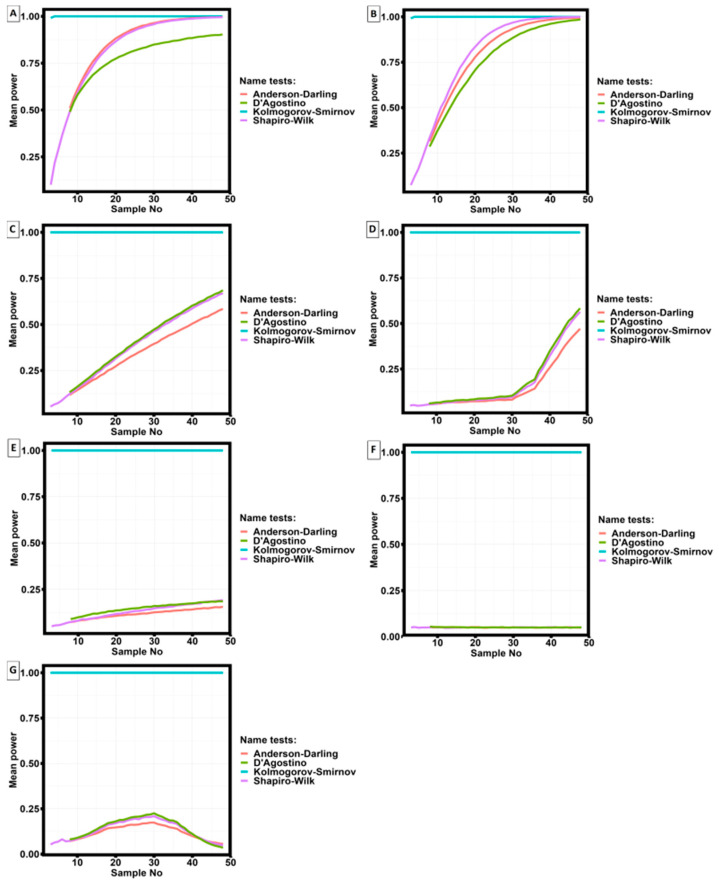
Dependence of the average power of a statistical criterion as a function of the number of trials for four statistical criteria and seven different distributions. (**A**) Cauchy distribution; (**B**) exponential distribution; (**C**) Gumbel distribution; (**D**) log-normal distribution; (**E**) logistic distribution; (**F**) Normal distribution; (**G**) Weibull distribution.

Figure 6 shows the results of calculating the average power of the statistical criterion depending on the number of trials.

Analysis of the results of calculations of the average statistical power of the four statistical criteria shows that the maximum power is possessed by the Kolmogorov–Smirnov criterion. The exception is the case when the measurement results obey the Cauchy distribution and the exponential distribution; when the number of trials is more than 40, the statistical power of the Anderson–Darling and Shapiro–Wilk criteria is almost equal to the power of the Kolmogorov–Smirnov criterion, and when the number of trials is more than 50, the power of the D’Agostino criterion approaches 1. For other distribution types, the statistical power of the Kolmogorov–Smirnov criterion is maximal. 

To determine the theoretical distribution closest to the data, two information criteria were applied: Akaike and Bayesian. The results of applying the Akaike and Bayesian criteria are presented in Table 6.

Thus, the dependence of the average statistical power of the criterion on the number of studies is reflected in Figure 6G, and the lowest probability of making an error of the second kind when analyzing the experimental results presented in this paper occurs when using the Kolmogorov–Smirnov criterion. 

Considering the results of modeling given in [55,56], the Kolmogorov–Smirnov criterion is the most applicable for data analysis in the problems of materials science, as it has the highest power and does not depend on the type of data distribution (in those cases when the closest type of data distribution are Weibull and Logistic distributions [56]). 

Using the Kolmogorov–Smirnov criterion, the data in Table 2 and Table 4 were tested for belonging to a normal distribution. Table 7 presents the results of the analysis.

Analysis of the results of applying the Kolmogorov–Smirnov test to surface roughness measurements and tensile test results show that the experimental values obtained do not belong to the normal distribution, and further statistical analysis should be carried out using non-parametric statistical criteria. 

Of practical interest are the correlations between surface roughness parameters and mechanical properties, the change in mechanical properties of samples made by selective laser melting technology and annealing temperature, and the behavior of surface roughness as a function of sample length. At the first stage of the analysis, point diagrams of the dependence of mechanical properties on surface roughness parameters were plotted.

**Figure 7:**
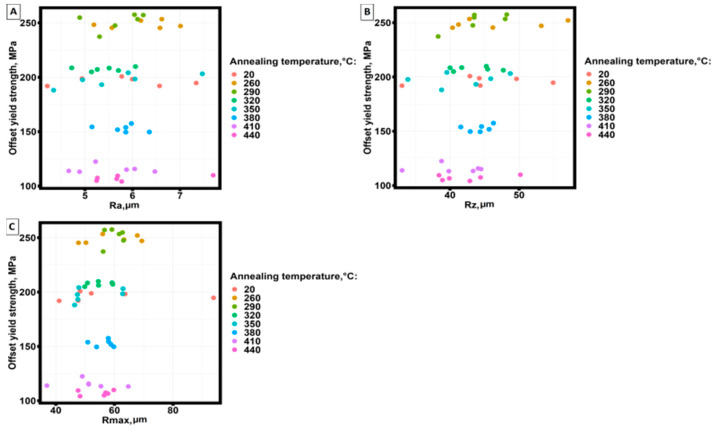
Dependence of yield strength on surface roughness parameters at different annealing temperatures of samples. (**A**) from Ra; (**B**) from Rz; (**C**) from Rmax.

Figure 7 shows an example of yield strength dependence on surface roughness parameters.

Analysis of the graphs (Figure 7) shows that the yield strength of samples made by selective laser melting technology from AlSi10Mg material practically does not change depending on the main parameters of surface roughness and has a clearly expressed division of data into groups depending on the annealing temperature. 

The behavior of the strength limit, strain corresponding to the yield strength, and strain corresponding to the strength limit depending on the main parameters characterizing the surface roughness did not show clearly expressed dependencies and stratification into groups.

The differences in the mechanical properties of the samples depending on the annealing temperature were analyzed using the Kruskal–Wallis criterion [57], the results of which are presented in Table 8.

The results of applying the Kruskal–Wallis criterion show that statistically significant differences are observed in the mechanical properties of samples obtained by selective laser melting technology annealed at different temperatures. No statistically significant differences were found in surface roughness parameters. A comparison of the test results (Table 8) with Figure 7 shows that mechanical properties do not have significant differences at all annealing temperatures. 

To test pairwise differences between mechanical properties depending on annealing temperature, the Mann–Whitney test was applied [58]. The results of the test are presented in Table 9.

The results of applying the Mann–Whitney criterion show that statistically significant differences are observed at almost all combinations of annealing temperatures, and all considered mechanical properties, except for aging temperatures 260 °C and 290 °C differences in all mechanical properties are not statistically significant. Except for yield strength, the same situation is observed at aging temperatures 320 °C and 350 °C; strength, strain corresponding to yield strength and strain corresponding to tensile strength have no statistically significant differences.

**Figure 8:**
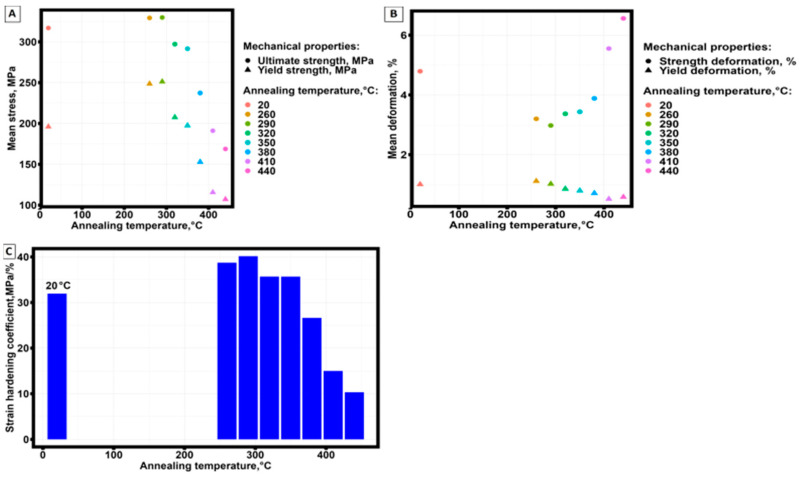
Dependence of average values of strength and yield strengths (**A**), strains corresponding to strength and yield strength (**B**), and strain hardening on the aging temperature of samples (**C**) obtained by selective laser melting technology from AlSi10Mg material.

Figure 8 shows the change in the average values of yield strength and tensile strength as a function of annealing temperature, strain corresponding to the tensile strength and yield strength, and the change in the strain hardening coefficient ($\theta =\frac{d\sigma }{d\varepsilon }$) [59] in the section of the tensile diagram from yield strength to tensile strength as a function of annealing temperature.

At an increase in aging temperature, there is a decrease in strength properties and an increase in the plasticity of samples obtained by selective laser melting technology from AlSi10Mg material (Figure 8A,B).

It follows from the presented dependences (Figure 8C) that the maximum strain hardening is achieved at the aging temperature equal to 290 °C. Considering the results of the analysis given in Table 9, the maximum strain hardening achieved is not statistically significantly different from the strain hardening achieved at 260 °C. 

**Figure 9:**
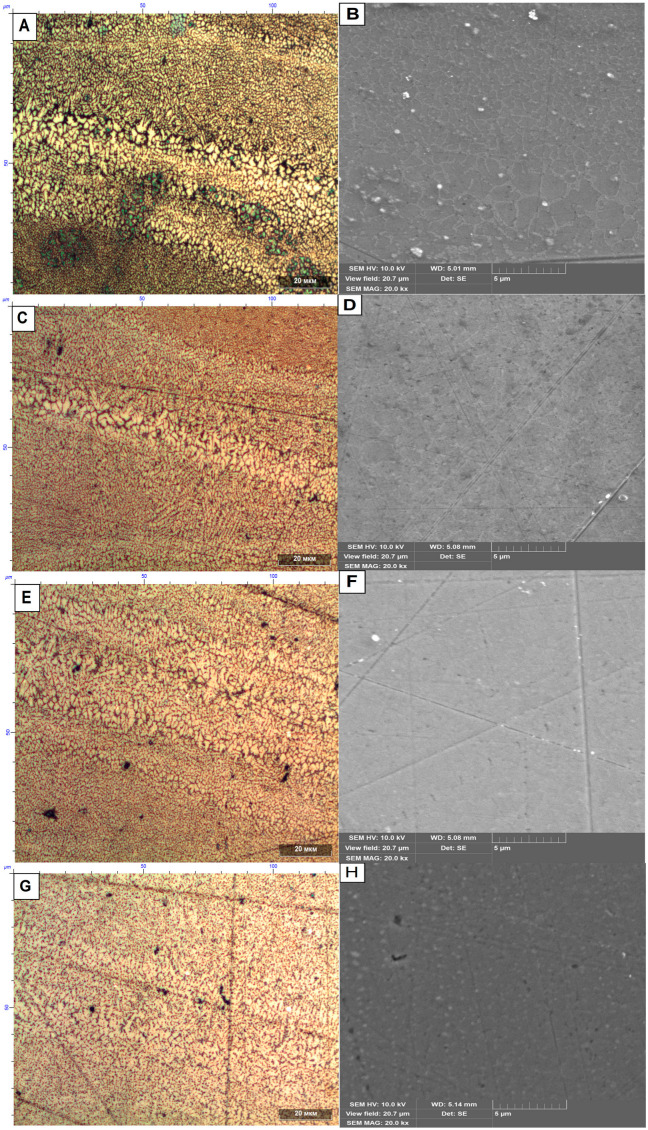
Microstructure of samples obtained by selective laser melting technology from AlSi10Mg material in the direction of laser motion and heat treated at (**A**) No heat treatment (Optical microscope); (**B**) No heat treatment (Scanning electron microscope); (**C**) heat treatment at 290 °C (Optical microscope); (**D**) heat treatment at 290 °C (Scanning Electron Microscope); (**E**) heat treatment at 380 °C (Optical Microscope); (**F**) heat treatment at 380 °C (Scanning Electron Microscope); (**G**) heat treatment at 440 °C (Optical Microscope); (**H**) heat treatment at 440 °C (Scanning Electron Microscope). (The smooth, straight lines in the figures are scratches left after polishing the samples).

The change of mechanical properties depending on the heat treatment temperature is associated with changes in the microstructure of the samples manufactured by SLM technology from AlSi10Mg material. Figure 9 shows the change in the microstructure of samples depending on the heat treatment temperature.

Metallographic analysis of the microstructure of the samples produced by SLM technology shows the presence of inhomogeneous microstructure across the width of the sample, which decreases with increasing heat treatment temperature. 

Figure 8A shows the microstructure of AlSi10Mg alloy produced by SLM without heat treatment. Three different types of grain structure are observed in it. In the middle part of the melt pool, a fine grain structure is obtained. In the lateral parts of the melt pool, the grains are larger and elongated towards the heat source, which corresponds to that described in [45]. A closer look at the microstructure of the samples without heat treatment on SEM shows a cellular microstructure [60], also having different cell sizes (Figure 8B).

Heat treatment of samples (Figure 9C) at 290 °C leads to a more uniform grain size distribution in the first and second zones from the melt center, while larger grains are retained at the melt boundary. Analysis of the microstructure of samples heat-treated at 290 °C with a scanning electron microscope (Figure 9D) shows a more uniform and less pronounced distribution of cells throughout the sample. With further heat treatment, the grain size becomes more uniform, and the cellular microstructure is no longer apparent (Figure 9E,F). When the heat treatment temperature is increased to 440 °C (Figure 9G), the microstructure becomes even more homogeneous, and inclusions of about 0.3 µm begin to appear on the samples.

**Figure 10:**
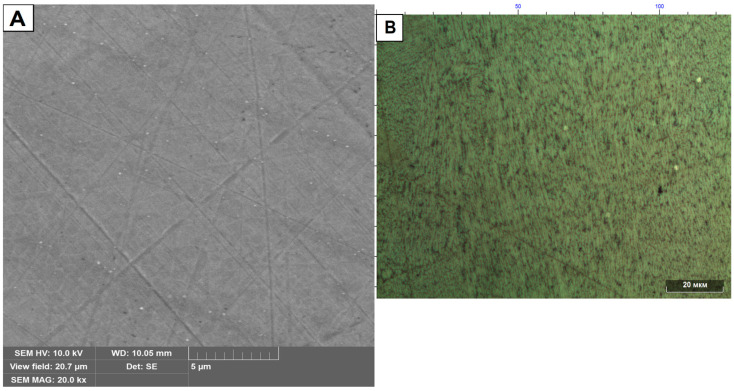
Microstructure of samples without heat treatment in cross-section with respect to laser motion. (**A**) Scanning electron microscope. (**B**) Optical microscope.

Figure 10 shows the microstructure of the samples without heat treatment in cross-section with respect to the laser motion.

Analysis of the microstructure of the cross-section of the sample shows the presence of grain structure (Figure 10B) and has a small number of inclusions (Figure 10A) similar in size to the inclusions presented in Figure 9H.

Thus, the maximum strain hardening of AlSi10Mg achieved at 290 °C can be attributed to the obtained sequential combination of fine and coarse-grained α-Al microstructure and the contribution of irregular eutectic phase varying with the heat treatment temperature [26,27,28,29].

To reveal not clearly expressed dependencies, correlation analysis of mechanical properties of samples obtained by selective laser melting technology from AlSi10Mg material and basic parameters describing surface roughness was applied. Considering the results of analyzing the distributions of the studied quantities (Table 6 and Table 7, the distribution is different from normal), the correlation analysis by Kendall was applied.

Table 10 shows the Kendall correlation coefficients, the calculated level of statistical significance, and the coefficient of determination. The strength of the correlation was interpreted using the Evans scale. The level of statistical significance was assumed to be 0.05.

The results of correlation analysis of mechanical properties of samples manufactured by selective laser melting technology from AlSi10Mg material and basic parameters of surface roughness show the presence of a weak statistically significant correlation between the strain corresponding to the yield strength and the sum of the height of the largest profile protrusion and the depth of the largest profile depression within the basic length of the sample (Rz) and between the strain corresponding to the yield strength and the full height of the profile (Rmax), in other cases statistically significant correlation between the strain corresponding to the yield strength and the full height of the profile (Rmax).

**Figure 11:**
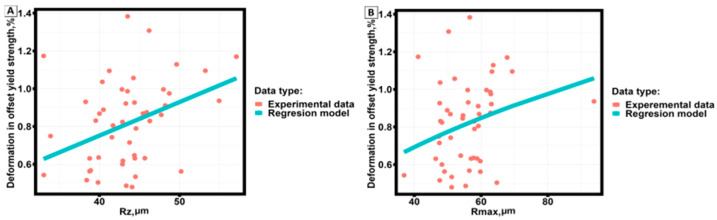
Dependence of strain corresponding to yield strength on (**A**) Rz, (**B**) Rmax, and regression models describing the dependence of correlated values.

Figure 11 shows scatter diagrams of the dependences of the strain corresponding to the yield strength as a function of Rz and Rmax and regression models describing the established dependences.

Table 11 presents the results of constructing the dependence of the strain corresponding to the yield strength on the surface roughness parameters.

The obtained correlations and regression equations describe a statistically significant relationship between the experimentally obtained data but do not provide an answer to the causes of the found relationship.

To establish the reasons for the correlation relationship, the sum of the heights of the largest protrusions and depths of the largest depressions of the surface roughness profile within the base length of the sample (Rz) and the total height of the surface roughness profile (Rmax) were analyzed. 

Rz is calculated by the equation:

(1) $$Rz=\frac{\sum_{i=1}^{5}\left|y_{pmi}\right|+\sum_{i=1}^{5}\left|y_{vmi}\right|}{5}$$

where $y_{pmi}$–height of the i-th protrusion of the surface roughness profile; $y_{vmi}$–depth of the i-th depression of the surface roughness profile.

Rmax, respectively:

(2) $$Rmax=|y_{max}-y_{min}|$$

where $y_{max}$–maximum height of roughness profile; $y_{min}$–maximum depth of surface roughness profile.

The analysis of the values included in Equations (1) and (2) shows that the main variables have extreme character, and their behavior should be analyzed by means of extreme value analysis [61]. However, it should be taken into account that the correlation is observed with the value characterizing the sample as a whole, and the analysis should be performed based on the influence of extreme values on each other. 

**Figure 12:**
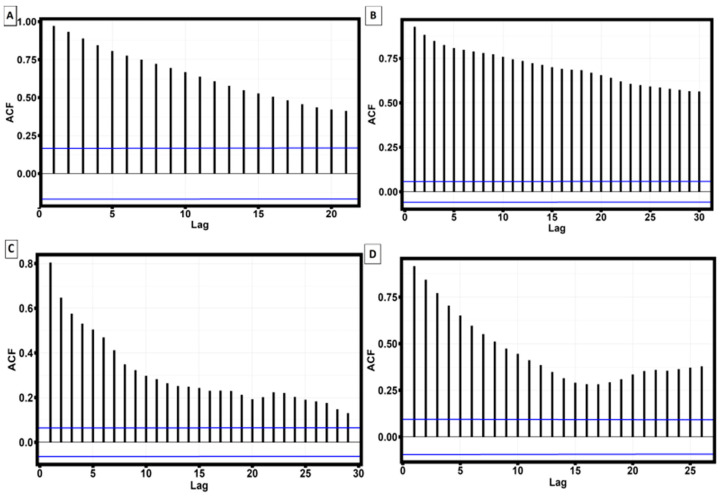
Autocorrelation functions of minima and maxima of the surface roughness profile. (**A**) ACF minima of sample No 4 aging at 440 °C; (**B**) ACF maxima of sample No 4 aging at 440 °C; (**C**) ACF minima of sample No 3 aging at 380 °C; (**D**) ACF minima of sample No 3 aging at 380 °C (The horizontal blue line indicates the level of statistical significance).

For these purposes, the autocorrelation function of the extreme values of the surface roughness profile was analyzed. As a result of the analysis, it was found that statistically significant autocorrelation of maxima and minima is observed only for two samples: sample No. 3, aged at 380 °C, and sample No. 4, aged at 440 °C. Figure 12 shows the graphs of autocorrelation functions of maxima and minima for these samples.

Removal of sample No. 3, aged at 380 °C, and sample No. 4, aged at 440 °C from the total sample, leads to the fact that the correlation between the strain corresponding to the yield strength and roughness parameters Rz and Rmax becomes statistically insignificant. Thus, the positive influence of surface roughness on the strain corresponding to the yield strength occurs when the maxima and minima of the surface roughness profile have a significant statistical correlation along the entire length of the sample.

## 4. Conclusions

As a result of statistical analysis of changes in mechanical properties and surface roughness depending on heat treatment, it was found that:

1. Maximum strain hardening of thin-walled samples made by selective laser melting technology from AlSi10Mg is achieved during the heat treatment for 1 h at 290 °C.
2. The mechanical properties of AlSi10Mg samples are not statistically significantly different at 260 °C and 290 °C.
3. The strain hardening of samples fabricated by SLM technology from AlSi10Mg is achieved due to the successive alternation of fine and coarse-grained α-Al microstructure and a more uniform distribution of the eutectic phase in the α-Al grain circle.
4. At heat treatment of samples in the temperature range from 290 °C to 440 °C within one hour, there are no statistically significant changes in surface roughness.
5. The correlation between the deformation corresponding to the yield strength and the sum of heights of the largest protrusions and depths of the largest depressions of the surface roughness profile within the basic length of the sample (Rz) and the full height of the surface roughness profile (Rmax) has been established.
6. The reason for the correlation is the stationary behavior of the maxima and minima of the surface roughness profile along the entire length of the specimens.

Summarizing the results of the study, we can conclude that heat treatment of thin-walled samples made by SLM technology from AlSi10Mg for 1 h at a temperature of 290 °C allows to achieve strain hardening of samples due to the successive alternation of fine-grained and coarse-grained microstructure surrounded by eutectic phase. The application of statistical analysis methods has shown that surface roughness has a positive effect on mechanical properties only under the condition of stationary behavior of maxima and minima of the surface roughness profile (the frequency and height of extreme values of the profile are unambiguously described by their average value and spectral function) along the entire length of the specimen. In other cases, surface roughness has no statistically significant relationship with mechanical properties under tension. 

During the study of the microstructure of the samples, the presence of inclusions with an average size of about 0.3 µm and anomalous behavior of the lattice parameter α-Al depending on the temperature of heat treatment were revealed. The study of these anomalies, as well as further studies of heat treatment modes and optimization of technological modes of manufacturing samples using SLM technology, will be the subject of further research.

## Figures and Tables

**Table 1:** Average chemical composition of AlSi10Mg powder.

Elements	Al	Si	Mg	O
Composition (wt.%)	88.1850	9.9550	0.3275	1.5325

**Table 2:** Surface roughness of samples produced by selective laser melting technology from AlSi10Mg.

No. of Samples	Annealing Temperature, °C	Ra, µm	Rz, µm	Rmax, µm
1	20	4.208	33.040	41.141
2	20	5.996	49.628	63.651
3	20	7.340	54.939	93.837
4	20	6.569	44.376	47.602
5	20	4.939	44.248	52.086
6	20	5.773	42.879	48.261
1	260	6.578	40.366	47.733
2	260	5.568	46.201	50.272
3	260	7.009	53.217	69.349
4	260	6.175	57.088	67.814
5	260	5.188	41.231	63.253
6	260	6.616	42.818	55.988
1	290	6.227	43.522	56.607
2	290	4.890	43.501	62.743
3	290	6.042	48.215	59.128
4	290	5.632	43.301	63.093
5	290	5.307	38.282	56.144
6	290	6.106	47.990	61.618
1	320	5.258	45.442	59.432
2	320	5.509	40.011	50.797
3	320	5.137	40.503	49.863
4	320	5.704	47.719	54.580
5	320	6.063	45.255	54.490
6	320	4.721	41.693	59.186
1	350	4.949	33.899	47.363
2	350	4.340	38.783	46.409
3	350	5.909	39.543	47.841
4	350	7.470	48.722	62.929
5	350	5.351	43.755	47.505
6	350	6.051	45.896	62.821
1	380	5.147	44.567	58.023
2	380	5.685	45.664	58.818
3	380	5.858	41.558	50.893
4	380	5.859	44.367	53.910
5	380	5.977	46.287	57.900
6	380	6.354	42.925	59.778
1	410	4.659	33.058	36.935
2	410	5.223	38.778	49.032
3	410	6.045	44.076	51.182
4	410	4.886	39.830	64.734
5	410	6.471	43.348	55.387
6	410	5.874	44.498	51.197
1	440	5.773	42.879	48.261
2	440	5.245	38.904	56.476
3	440	5.261	44.417	56.988
4	440	5.688	38.412	47.601
5	440	7.698	50.192	59.783
6	440	5.668	39.901	57.787

**Table 3:** Basic statistical analysis of the results of surface roughness measurements of 48 samples manufactured by selective laser melting technology from AlSi10Mg.

Statistical Parameter	Annealing Temperature, °C	Ra, µm	Rz, µm	Rmax, µm
Mean value, µm	20	5.804	44.852	57.763
Median, µm		5.885	44.312	50.174
Standard deviation, µm		1.121	7.329	19.173
Maximum value, µm		7.34	54.939	93.837
Minimum value, µm		4.208	33.04	41.141
Mean value, µm	260	6.189	46.820	59.068
Median, µm		6.377	44.510	59.621
Standard deviation, µm		0.692	6.865	9.111
Maximum value, µm		7.009	57.088	69.349
Minimum value, µm		5.188	40.366	47.733
Mean value, µm	290	5.701	44.135	59.889
Median, µm		5.837	43.511	60.373
Standard deviation, µm		0.524	3.667	3.059
Maximum value, µm		6.227	48.215	63.093
Minimum value, µm		4.89	38.282	56.144
Mean value, µm	320	5.399	43.437	54.725
Median, µm		5.384	43.474	54.535
Standard deviation, µm		0.468	3.132	4.030
Maximum value, µm		6.063	47.719	59.432
Minimum value, µm		4.721	40.011	49.863
Mean value, µm	350	5.678	41.766	52.478
Median, µm		5.63	41.649	47.673
Standard deviation, µm		1.080	5.388	8.068
Maximum value, µm		7.47	48.722	62.929
Minimum value, µm		4.34	33.899	46.409
Mean value, µm	380	5.813	44.228	56.554
Median, µm		5.859	44.467	57.962
Standard deviation, µm		0.396	1.747	3.421
Maximum value, µm		6.354	46.287	59.778
Minimum value, µm		5.147	41.558	50.893
Mean value, µm	410	5.526	40.598	51.411
Median, µm		5.549	41.589	51.190
Standard deviation, µm		0.712	4.373	9.040
Maximum value, µm		6.471	44.498	64.734
Minimum value, µm		4.659	33.058	36.935
Mean value, µm	440	5.889	42.451	54.483
Median, µm		5.678	41.39	56.732
Standard deviation, µm		0.915	4.458	5.202
Maximum value, µm		7.698	50.192	59.783
Minimum value, µm		5.245	38.412	47.601

**Table 4:** Basic mechanical properties of samples obtained by selective laser melting from AlSi10Mg material.

No. of Samples	Annealing Temperature, °C	σ_0.2_, MPa	σ_U_, MPa	ε_0.2_, %	ε_U_, %
1	20	191.948	308.964	1.173	4.998
2	20	198.216	310.750	1.129	4.478
3	20	194.706	313.314	0.936	4.406
4	20	192.044	314.810	0.928	4.988
5	20	198.825	326.616	1.057	5.222
6	20	200.728	327.219	0.824	4.653
1	260	245.334	328.600	1.036	3.136
2	260	245.452	334.828	1.308	3.919
3	260	247.062	328.213	1.095	3.261
4	260	252.070	321.049	1.169	2.735
5	260	248.233	327.996	1.095	3.170
6	260	253.389	334.916	0.996	2.981
1	290	257.103	333.964	1.383	3.406
2	290	254.805	334.178	0.986	2.791
3	290	257.494	341.998	0.912	2.903
4	290	247.495	328.838	0.922	2.879
5	290	237.288	316.883	0.931	3.025
6	290	253.389	322.998	0.996	2.862
1	320	207.151	294.796	0.869	3.175
2	320	208.406	293.716	0.868	3.074
3	320	204.945	296.520	0.888	3.706
4	320	206.205	298.721	0.862	3.776
5	320	209.819	298.073	0.845	3.136
6	320	208.615	301.139	0.805	3.343
1	350	197.684	283.684	0.749	2.640
2	350	188.093	288.349	0.631	3.194
3	350	204.146	301.434	0.831	3.839
4	350	203.145	288.471	0.975	3.559
5	350	193.197	294.468	0.715	3.710
6	350	198.387	293.003	0.875	3.666
1	380	154.363	235.000	0.789	3.926
2	380	151.798	238.317	0.632	3.744
3	380	153.899	236.487	0.743	3.759
4	380	149.531	235.273	0.647	3.821
5	380	157.501	239.803	0.829	4.186
6	380	149.708	239.161	0.618	3.865
1	410	113.933	186.648	0.543	5.369
2	410	122.491	201.566	0.563	5.230
3	410	115.744	190.155	0.479	4.499
4	410	113.202	186.602	0.504	5.130
5	410	113.399	190.513	0.486	6.981
6	410	115.118	191.084	0.534	6.101
1	440	104.216	164.398	0.600	6.432
2	440	104.979	164.362	0.567	6.293
3	440	107.472	169.422	0.632	7.125
4	440	109.452	173.549	0.516	6.747
5	440	109.941	173.112	0.562	6.346
6	440	106.627	167.986	0.635	6.415

**Table 5:** Basic statistical analysis of tensile test results of 48 specimens fabricated by selective laser melting technology from AlSi10Mg.

Statistical Parameter	Annealing Temperature, °C	σ_0.2_, MPa	σ_U_, MPa	ε_0.2_, %	ε_U_, %
Mean value	20	196.078	316.946	1.008	4.791
Median		196.461	314.062	0.996	4.821
Standard deviation		3.713	7.986	0.134	0.326
Maximum value		200.728	327.219	1.173	5.222
Minimum value		191.948	308.964	0.824	4.406
Mean value	260	248.590	329.267	1.117	3.200
Median		247.648	328.407	1.095	3.153
Standard deviation		3.407	5.168	0.111	0.397
Maximum value		253.389	334.916	1.308	3.919
Minimum value		245.333	321.049	0.996	2.735
Mean value	290	251.262	329.810	1.021	2.978
Median		254.097	331.401	0.958	2.891
Standard deviation		7.739	8.937	0.181	0.223
Maximum value		257.494	341.998	1.383	3.406
Minimum value		237.288	316.883	0.911	2.791
Mean value	320	207.524	297.161	0.856	3.368
Median		207.779	297.297	0.865	3.259
Standard deviation		1.776	2.719	0.029	0.303
Maximum value		209.819	301.139	0.888	3.776
Minimum value		204.945	293.716	0.805	3.074
Mean value	350	197.442	291.568	0.796	3.434
Median		198.035	290.737	0.790	3.612
Standard deviation		6.064	6.163	0.123	0.447
Maximum value		204.146	301.434	0.975	3.839
Minimum value		188.093	283.684	0.631	2.640
Mean value	380	152.800	237.340	0.710	3.884
Median		152.849	237.402	0.695	3.843
Standard deviation		3.066	2.040	0.090	0.163
Maximum value		157.501	239.803	0.829	4.186
Minimum value		149.531	235	0.618	3.744
Mean value	410	115.648	191.095	0.518	5.552
Median		114.525	190.334	0.519	5.300
Standard deviation		3.496	5.492	0.034	0.868
Maximum value		122.491	201.566	0.563	6.981
Minimum value		113.202	186.602	0.479	4.499
Mean value	440	107.115	168.805	0.585	6.560
Median		107.050	168.704	0.584	6.424
Standard deviation		2.314	4.032	0.046	0.319
Maximum value		109.941	173.549	0.635	7.125
Minimum value		104.216	164.362	0.516	6.293

**Table 6:** Closest distribution types according to the minimum of Akaike and Bayesian criteria.

Physical Parameter	Closest Type of Distribution
σ_0.2_, MPa	Weibull
σ_U_, MPa	Weibull
ε_0.2_, %	Weibull
ε_U_, %	Weibull
Ra, µm	Log-normal
Rz, µm	Logistical
Rmax, µm	Logistical

**Table 7:** Results of testing whether the data in Table 2 and Table 4 belong to a normal distribution.

Kolmogorov–Smirnov Statistics	σ_0.2_, MPa	σ_U_, MPa	ε_0.2_, %	ε_U_, %	Ra, µm	Rz, µm	Rmax, µm
D	1	1	0.68409	0.99585	0.99999	1	1
*p*-value	<2.2 × 10^−16^	8.9 × 10^−16^	<2.2 × 10^−16^	8.9 × 10^−16^	<2.2 × 10^−16^	<2.2 × 10^−16^	<2.2 × 10^−16^

**Table 8:** Results of applying the Kruskal–Wallis criterion to the data given in Table 2 and Table 4 in the study of the influence of annealing temperature on mechanical properties and surface roughness characteristics.

Investigated Quantity	Statistical Significance Level by Kruskal–Wallis Test
σ_0.2_, MPa	1.18 × 10^−7^
σ_U_, MPa	1.44 × 10^−7^
ε_0.2_, %	9.89 × 10^−7^
ε_U_, %	9.95 × 10^−7^
Ra, µm	0.72
Rz, µm	0.67
Rmax, µm	0.43

**Table 9:** Results of the analysis of statistical differences in groups of samples aged at different temperatures.

Annealing Temperature Pairs	Results of Applying the Mann–Whitney Criterion for Mechanical Properties of Samples			
	σ_0.2_, MPa	σ_U_, MPa	ε_0.2_, %	ε_U_, %
20–260	0.002165	0.008658	0.3095	0.002165
20–290	0.002165	0.02597	0.8182	0.002165
20–350	0.6991	0.002165	0.04113	0.002165
260–290	0.1994	0.9372	0.07765	0.3095
260–320	0.002165	0.002165	0.002165	0.3939
260–350	0.002165	0.002165	0.002165	0.3939
290–350	0.002165	0.002165	0.01515	0.09307
320–350	0.002165	0.09307	0.3939	0.5887
350–380	0.002165	0.002165	0.2403	0.01515

**Table 10:** Results of Kendall correlation analysis between the main mechanical properties and surface roughness parameters of the samples obtained by selective laser melting technology from AlSi10Mg material.

Pairs Examined for Correlation	Kendall Correlation Coefficient	Statistical Significance Level	Determination Coefficient, %
σ_0.2_–Ra	0.2822	0.3282	--
σ_U_–Ra	0.1073171	0.2822	--
ε_0.2_, %–Ra	0.1259982	0.2069	--
ε_U_, %–Ra	0.09760426	0.3282	--
σ_0.2_–Rz	0.1774623	0.07545	--
σ_U_–Rz	0.1676275	0.09298	--
ε_0.2_, %–Rz	0.2342502	0.01894	5.5
ε_U_, %–Rz	−0.06829269	0.4937	--
σ_0.2_–Rmax	0.1792369	0.07257	--
σ_U_–Rmax	0.1268293	0.2037	--
ε_0.2_, %–Rmax	0.2040816	0.04091	4.2
ε_U_, %–Rmax	−0.1764967	0.07693	--

**Table 11:** Equations describing a weak correlation between the strain corresponding to yield strength and surface roughness parameters.

Correlation Values		Equations	Standard Deviation
ε_0.2_	Rz	$\varepsilon_{0.2}=0.0406+0.0178\times z$	0.1812
	Rmax	$\varepsilon_{0.2}=0.0033+0.1090\times \sqrt[{2}]{Rmax}$	0.2347

## Data Availability

Data are contained within the article.
